# Simultaneous EEG-NIRS Measurement of the Inferior Parietal Lobule During a Reaching Task With Delayed Visual Feedback

**DOI:** 10.3389/fnhum.2019.00301

**Published:** 2019-09-06

**Authors:** Takuro Zama, Yoshiyuki Takahashi, Sotaro Shimada

**Affiliations:** ^1^Electrical Engineering Program, Graduate School of Sciences and Technology, Meiji University, Kawasaki, Japan; ^2^Department of Electronics and Bioinformatics, School of Sciences and Technology, Meiji University, Kawasaki, Japan

**Keywords:** electroencephalography, near-infrared spectroscopy, simultaneous measurement, psychophysiological interaction, event-related (de)synchronization, inferior parietal lobule, visual feedback delay

## Abstract

We investigated whether the inferior parietal lobule (IPL) responds in real-time to multisensory inconsistency during movement. The IPL is thought to be involved in both the detection of inconsistencies in multisensory information obtained during movement and that obtained during self-other discrimination. However, because of the limited temporal resolution of conventional neuroimaging techniques, it is difficult to distinguish IPL activity during movement from that during self-other discrimination. We simultaneously conducted electroencephalography (EEG) and near-infrared spectroscopy (NIRS) with the goal of examining IPL activity with a high spatiotemporal resolution during single reaching movements. Under a visual feedback-delay condition, gamma event-related synchronization (γ-ERS), i.e., an increase in gamma (31–47 Hz) EEG power occurred during reaching movements. This γ-ERS is considered to reflect processing of information about prediction errors. To integrate this temporal information with spatial information from the NIRS signals, we developed a new analysis technique that enabled estimation of the regions that show a hemodynamic response characterized by EEG fluctuation present in the visual feedback-delay condition. As a result, IPL activity was explained by γ-ERS specific to visual feedback delay during movements. Thus, we succeeded in demonstrating real-time activation of the IPL in response to multisensory inconsistency. However, we did not find any correlation between either IPL activity or γ-ERS with the sense of agency. Therefore, our results suggest that while the IPL is influenced by prediction error signals, it does not engage in direct processing underlying the conscious experience of making a movement, which is the foundation of self-other discrimination.

## Introduction

When watching a moving hand, we are easily able to determine whether the hand is our own or belongs to someone else based on the consistency between the action and our proprioceptive sensation. The inferior parietal lobule (IPL) is thought to play an important role in this essential ability to discriminate between the self and others. Injury to the IPL has been found to affect the ability to judge whether a hand movement observed on a monitor is performed by the observer or by another person (Sirigu et al., 1999). Furthermore, previous neuroimaging studies of healthy participants have indicated that IPL activation increases (particularly in the right hemisphere) in response to temporal or spatial errors in visual feedback about movements (Farrer and Frith, 2002; Farrer et al., 2003, 2008; Shimada et al., 2005; Balslev et al., 2006). Based on these findings, researchers have proposed that the right IPL is involved in attributing actions to others based on multisensory inconsistencies. In describing the self-other recognition process, Synofzik et al. (2008) proposed a two-step account of agency in which an individual first experiences a “feeling of agency” and then a “judgment of agency.” The feeling of agency is a lower-level perceptual representation that refers to an online sense of control based on sensory feedback and proprioception. In contrast, the judgment of agency is a higher-level conscious and retrospective judgment that attributes agency based on factors such as the feeling of agency, intention, and social cues. Although recent neuroimaging studies have indicated that the IPL plays an important role in the contribution of agency, whether the IPL contributes to the “feeling of agency” or the “judgment of agency” is unclear. The low temporal resolution of the imaging techniques used in previous studies has prevented the separation of brain activity related to the real-time feeling of control from that related to conscious judgment of agency.

Recent neuroimaging studies have indicated that multimodal simultaneous measurement will improve the spatiotemporal resolution of brain imaging (Shibasaki, 2008; Bießmann et al., 2011). Among the existing human neuroimaging techniques, some have higher spatial resolution and others have higher temporal resolution. Typical techniques such as functional magnetic resonance imaging (fMRI) and near-infrared spectroscopy (NIRS) have higher spatial and lower temporal resolution, while electroencephalography (EEG) and magnetoencephalography (MEG) have lower spatial and higher temporal resolution. The integration of multiple measurement strategies has the potential to maximize both spatial and temporal information. In particular, the coupling of EEG and NIRS is suitable for simultaneous measurement because these two methods can be used to measure signals derived from brain activity without interfering with one another. In the case of EEG and fMRI, the different signals can contaminate one another such that considerable effort and sophisticated measurement techniques are necessary to confirm that the signals are truly reflecting brain activity. Recent studies using simultaneous EEG-NIRS measurement have shown that fluctuations in EEG and NIRS signals are correlated (Horovitz and Gore, 2004; Takeuchi et al., 2009; Näsi et al., 2010; Zama and Shimada, 2015). Takeuchi et al. (2009) investigated the correlation between EEG somatosensory-evoked potential (SEP) and NIRS signals from the somatosensory area. They conducted general linear model (GLM) analysis (Friston et al., 1994) and reported that the correlation between NIRS signals and SEPs increased when they inserted an onset delay. They suggested that simultaneous EEG-NIRS measurement would be useful for revealing the temporal order of neural activation. However, they only used a correlation analysis to examine how the fluctuations in each signal were related. In the present study, we integrated physiological (EEG signals rich in time information) and psychological (experimental) factors in terms of the regional hemodynamic response (NIRS signal) to interpret the functional specificity of the parietal area for a multisensory inconsistency. This new technique can be used to estimate the activation area in which NIRS fluctuations will be described by EEG features that exhibit responses specific to an experimental factor (i.e., a multisensory inconsistency due to visual feedback delay).

Here, we simultaneously measured EEG and NIRS during a reaching movement with a visual feedback delay. We investigated whether the IPL would be affected in real-time by processing of multisensory inconsistencies reflected in EEG signals. The activity of IPL has been reported to occur during movement as a sensorimotor interface (Mattingley et al., 1998; Gao et al., 2011). If the IPL responds to multisensory inconsistency online during movement, it is assumed that the IPL will receive information about the multisensory inconsistency. Previous studies have reported that EEG gamma-band activity is involved not only in multisensory integration (Sakowitz et al., 2001, 2005; Senkowski et al., 2005; Kanayama et al., 2007, 2009; Schneider et al., 2008), but also the prediction error (Arnal et al., 2011; Arnal and Giraud, 2012). The error-related gamma oscillations have been suggested to involve with activity of anterior cingulate cortex (ACC), superior temporal sulcus (STS) and temporoparietal junction (TPJ) (van Pelt et al., 2016; Gillies et al., 2019). Further, EEG gamma oscillations have been found to explain fluctuations in fMRI signals (Engell et al., 2012; Magri et al., 2012; Mizuhara, 2012; Tagliazucchi et al., 2012). From the above, if the error-related information reflected in gamma oscillations is input to the IPL, it is expected that the activation pattern in the IPL under the visual feedback delay will be explained by the error-related gamma oscillation.

## Materials and Methods

### Participants

Sixteen healthy male volunteers (aged 22.3 ± 1.3 years, mean ± *SD*) participated in the experiment. All participants were right-handed (Chapman’s handedness inventory score: 14.1 ± 1.7 points; Chapman and Chapman, 1987) and had normal or corrected-to-normal visual acuity. We obtained written informed consent from all participants before the experiment. The experiment was approved by the ethics committee of the School of Science and Technology, Meiji University. We conducted the experiment according to the principles and guidelines of the Declaration of Helsinki.

One participant was removed from the analysis based on poor behavioral performance and another because of a noisy EEG signal (see section “Data Analysis”). Thus, we analyzed data from 14 of the 16 participants.

### Apparatus

**FIGURE 1:**
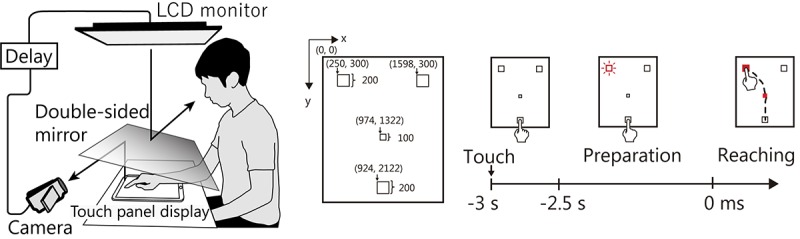
Experimental setup and task procedure. Participants performed a reaching task using a tough panel display (Zama et al., 2017). They observed a moving image of their right hand that was projected onto a mirror. In the Delay condition, the feedback image was delayed to modulate the sense of agency with respect to their hand. Participants were instructed to swipe their right index finger across the touch panel display from the home position to **left**/**right** goal areas via a central square. They were instructed to perform the movements as quickly and accurately as possible after seeing the “go” cue.

The participants sat on a chair and placed their right hand on a table (Figure 1) with a touch panel display (12.9 in, 2732 × 2048 resolution, 264 ppi, refresh rate 60 Hz; iPad Pro, Apple, United States). A double-sided mirror was positioned above the table such that the backside reflected the touch panel and the participants’ right hands onto a video camera (HDR-CX670, Sony, Japan). The recorded images were presented on a liquid–crystal monitor (LMD-232W, SONY, Japan) that was set above the front face of the mirror. Thus, participants could see the touch panel display and their hand on the mirror. The angle of the mirror was precisely adjusted before starting the experiment to make the touch panel display appear as if it was positioned horizontally on the desk. A time delay device (EDS3305, Eletex, Japan) was connected between the video camera and the LCD monitor. The delay could be inserted in 33-ms steps, which corresponds to 1 video frame. We used a high-speed camera (EX-F1, CASIO, Japan) to determine that the default (minimum) feedback delay was at least approximately 121 ms.

### Procedure

Participants performed a swiping movement with their right index finger from the home position to either a left- or right-side goal area displayed on the touch screen (Figure 1). This swiping movement is a form of “reaching” that is performed based on visual information. When the participants tapped the home position, the border of one of the goal areas turned red, cuing the target location and allowing participants to prepare their reaching movements. After a 2.5-s preparation period, the central square and goal became red (the “go” cue), signaling that the participant should move their fingers. The participants then began reaching toward the goal position as quickly and accurately as possible, taking care to pass their finger through the central square. Note that participants were instructed to fixate on the central square until the go cue was presented and to not make any extra movements once they stopped the reaching movement, even if their fingers had not reached the goal. To avoid providing extra clues regarding the judgment of agency, finger trajectory was not drawn on the touch screen. To reduce the contamination of EEG and NIRS signals by motion artifacts, participants were instructed to put their chin on the chin rest and to move their body parts as little as possible, except the right arm, during the task.

Each experimental session comprised two blocks containing 60 trials with a 3-min inter-block interval (total 120 trials). Within each block, visual Delay (50%) and Non-Delay (50%) trials were presented pseudorandomly. In the Delay condition, the visual feedback delay was 319 ms. This length was achieved by adding 6 frames to the default delay (121 ms in the Non-delay condition). The delay lengths were determined based on the delay-detection threshold that we reported in a previous study: in a similar reaching task, participants began to notice the delay when it exceeded 220 ms (3 delay-frames greater than the default delay) (Zama et al., 2017). Thus, for the present study, we set the conditions such that the delay would be hardly noticeable or easily noticeable. After each trial, the participants reported whether they thought the observed hand image had been completely synchronized with their own movement (i.e., whether the visual feedback was delayed or not).

After completion of the main trials, participants filled out a questionnaire that examined the relationship between the psychological recorded data and the subjective experience of agency, or “sense of agency” (SoA). This sense is best understood as the feeling that an “action was caused by myself” (Gallagher, 2000). Participants performed 4 additional trials (2 conditions × 2 trials, random order) and filled out the questionnaire (Kalckert and Ehrsson, 2012) after each reaching movement. They indicated the extent of their agreement or disagreement with the following four statements: (1) “The observed hand moved just like I wanted it to, as if it was obeying my will.” (2) “I felt as if I was controlling the movements of the observed hand.” (3) “I felt as if I was causing the movement I saw.” (4) “Whenever I moved my hand, I expected the observed hand to move in the same way.” Every question was rated on a seven-point Likert-like scale ranging from “−3” (totally disagree) to “+ 3” (totally agree), with “0” indicating neither agreement nor disagreement.

### Measurement of Brain Function

#### EEG-NIRS Simultaneous Measurement

**FIGURE 2:**
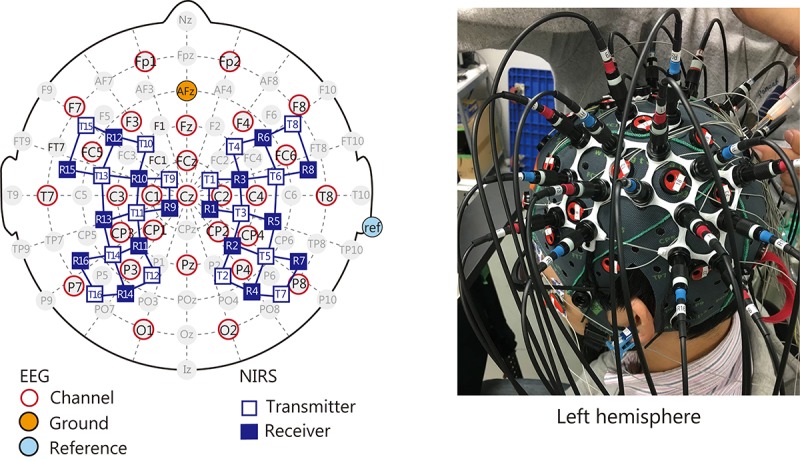
Location of EEG electrodes and NIRS optodes arranged in the customized cap. Both sensors were placed over the frontoparietal areas that are important for visuomotor function.

Electroencephalography electrodes and NIRS optodes were arranged in a customized EEG-NIRS cap (Figure 2). EEG data were recorded from 28 channels based on the extended 10–20 system (Fp1, Fp2, F7, F3, Fz, F4, F8, FC5, FCz, FC6, T7, C3, C1, Cz, C2, C4, T8, CP3, CP1, CP2, CP4, P7, P3, Pz, P4, P8, O1, and O2). NIRS data were recorded from 22 channels over the fronto-parietal area of both hemispheres, as this region has been reported to be involved in making reaching movements (Rizzolatti and Luppino, 2001).

#### EEG Configuration

Electroencephalography data were recorded using a 24-bit biosignal amplification unit (g.USBamp, g.tec Medical Engineering GmbH, Austria) at a sampling frequency of 1200 Hz with a 100-Hz anti-aliasing filter. The signals were recorded with active Ag/AgCl electrodes. The ground electrode was located on the forehead (AFz of the extended 10–20 system) and the reference was mounted on the right earlobe. We also recorded vertical and horizontal electrooculographic (EOG) data with the same amplification unit.

#### NIRS Configuration

Hemodynamic responses were recorded using a multichannel NIRS unit operating at wavelengths of 780, 805, and 830 nm (OMM-3000, Shimadzu, Japan) with a sampling frequency of 10 Hz. NIRS assesses hemodynamic responses as temporal changes in oxyhemoglobin (oxyHb) and deoxyhemoglobin (deoxyHb) concentrations, as well as total hemoglobin (totalHb).

The optode locations were measured using a 3-D magnetic space digitizer (Fastrak, Polhemus, United States). The measured position data were processed via a probabilistic spatial registration method (Lancaster et al., 2000; Singh et al., 2005). This method enabled us to register NIRS channel locations to the standard Montreal Neurological Institute (MNI) – 152 compatible canonical brain, which has been optimized for NIRS analysis through the 10–20 system, instead of using MRI datasets from individual participants. In this study, the standard deviations for registration on the cortical surface were under 10 mm. Thus, we assessed hemodynamic responses according to changes in concentrations of oxyHb, deoxyHb, and totalHb at each NIRS channel, which was registered to MNI space.

### Data Analysis

#### Delay Detection

Previous studies have shown that the threshold for a noticeable delay in visual feedback is around 220 ms (Zama et al., 2017). Therefore, we were confident that most participants would notice the 319-ms delay that we used in this experiment. Even so, we verified this using a questionnaire. We estimated that the questionnaire data would have a binomial distribution such that participants would report the delay in 55.1 ± 4.5 (mean ± *SD*) and 2.6 ± 2.5 of the 60 trials in the Delay and Non-delay conditions, respectively. One participant who did not notice the delay in 80% of the Delay trials was removed from the analysis as an outlier.

#### Behavioral Performance

We analyzed the 2-D trajectory data obtained from the touch screen. Movement duration was defined according to a velocity threshold of 10 mm/s (Messier and Kalaska, 1999; Igarashi et al., 2011). Motor performance was evaluated according to the trajectory error and the endpoint error (see Zama et al., 2017 for details). Trajectory error was defined as the degree of deviation from the ideal trajectory drawn in the Non-delay condition. Endpoint error was defined as the Euclidean distance from the center of the goal area to the endpoint in each trial. We evaluated the conscious experience of motion control as the mean score on the SoA questionnaire. These indexes were compared between conditions using one-sample *t*-tests.

#### Time-Frequency Analysis for EEG Data

Electroencephalography data were preprocessed with a 0.1 Hz high-pass filter and a 47 Hz low-pass filter, and then resampled at 200 Hz. The filters were zero-phase. To eliminate ocular artifacts, we conducted an independent components analysis of the EEG data. We rejected two independent components that were most significantly correlated with the vertical and horizontal EOG (*r*^2^ > 0.16). The remaining data were back-projected to EEG channels and segmented into 1.6-s epochs beginning after go-cue presentation (on average, the reaching task lasted 1.4 s from the go cue). Epochs exceeding a ± 150 μV amplitude criterion over the majority of the electrodes were considered contaminated by motion artifacts and thus excluded from further analysis. Of the 60 trials in each condition, the numbers of trials remaining after these exclusions were 57.9 ± 2.0 for the Delay condition and 57.1 ± 6.2 for the Non-delay condition. One participant was excluded from subsequent analyses because only 34 Delay trials remained after the exclusion process.

Finally, we conducted time-frequency analysis with a wavelet transformation. The wavelet transform signal was acquired by convolving the EEG signal with a complex Morlet’s wavelet function *w*(*t*, *f*):

$$w\,\left(t,f\right)={\left(\sigma_{t}\,\sqrt{\pi }\right)}^{-\frac{1}{2}}\,e\,x\,p\,\left(-\frac{t^{2}}{2\,\sigma_{t}^{2}}\right)\,e\,x\,p\,\left(j\,2\,\pi \,f\,t\right)$$

where σ_*t*_ is a standard deviation of a Gaussian window. The wavelet is characterized by the number of cycles (n_*co*_) within the Gaussian window (Lachaux et al., 2000). In this study, we set *n*_*co*_ = 6, with the frequency *f* ranging from 4 to 47 Hz in 1-Hz steps. The power of each frequency was calculated and averaged across trials to investigate event-related (de)synchronization (ERS/ERD), which is a phenomenon in which EEG power increases (decreases) in relation to an event. The averaged power values were normalized to a baseline that was set to the time-bin occurring 0.5 s after the go cue. The statistical analysis for the average power-value was conducted using a one-sample two-tailed *t*-test within a sliding-window with a length that corresponded to two cycles of the target frequency. The sliding step was 0.2 s. False discovery rate (FDR) correction (Benjamini and Hochberg, 1995) was applied based on step number for every target frequency.

#### Cross-Frequency Coupling Analysis for EEG Data

We also analyzed the synchrony of EEG oscillations to investigate whether the error-related information reflected in gamma oscillations would be input to the parietal area. It is well known that the alpha (8–13 Hz) power decreases around central area during movement. This motor-related ERD at alpha band (α-ERD) around central area has been dubbed “mu-suppression,” and is considered to be related to processing in the sensorimotor system (Pfurtscheller and Neuper, 1994). With regard to reaching movements, the motor-related α-ERD has been reported to occur around parietal region contralateral to the performing hand (Fumuro et al., 2015). If the information processing reflected by the γ-ERS is integrated into the parietal lobe, the γ-rhythm oscillation is expected to show a coupling with the motor-related alpha-rhythm oscillation.

To identify the relation between phases of alpha and gamma rhythms within an electrode, we analyzed the phase-phase coupling (PPC) at the time bin at which the significant γ-ERS occurred. The PPC is defined by following equation:

$$P\,P\,C\,\left(f_{1},f_{2}\right)=\frac{1}{N}\,\left|\sum_{i=1}^{N}e^{j\,\left(m\times \varphi_{\left(i,f_{1}\right)}-\varphi_{\left(i,f_{2}\right)}\right)}\right|$$

where φ_*(i,f*_1_)_ is the phase of *f*_1_ at the time point *i*, and *f*_1:_ *f*_2_ = 1: *m* (Kawasaki et al., 2010). In the present study, the remarkable γ-ERS occurred at 39 Hz, so we analyzed coupling between 13 Hz and 39 Hz. The PPCs were averaged across trials for each Delay and Non-Delay condition. To evaluate visual feedback-delay related change in PPC, we applied permutation test for the PPCs between Delay and Non-Delay conditions (i.e., after randomizing PPCs across conditions and rerunning the statistical test 2000 times, we compared the distribution of that statistical values and the original statistical value calculated with the original data set) (Cohen, 2014; Hülsemann et al., 2019). The statistical comparison was done with *z*-value from the two-sided Wilcoxon signed rank test.

To identify the relation between phase of alpha-rhythm and amplitude of gamma rhythm within an electrode, we analyzed the phase-amplitude coupling (PAC) (Canolty et al., 2006; Canolty and Knight, 2010; Kajihara et al., 2015; Hülsemann et al., 2019) at the time bin at which the significant γ-ERS occurred. We calculated modulation index (MI) which reflects the strength of coupling. The MI is based on the following complex variable:

$$z\,\left(t\right)=A_{1}\,\left(t\right)\,e^{j\,\varphi_{2}\,\left(t\right)}$$

where *A*_1_(t), and φ_2_(*t*) represent the amplitude of a higher frequency (gamma), and the phase of another lower frequency (alpha), respectively. The MI is the absolute mean vector of the complex variable as follows:

$$M\,I=\frac{1}{T}\,\left|\sum_{t=1}^{T}z\,\left(t\right)\right|$$

The MI was calculated on each trial and normalized to the distribution of surrogate data to correct for the different amplitudes across trials. Surrogate data ware generated by calculating MI between the original phase time-series and a permuted amplitude time-series (Hülsemann et al., 2019). The permuted amplitude time-series was constructed by cutting the original one at a random time point and swapping the order of the separated parts. The shuffling was repeated 1000 times. The observed original MI was normalized by the mean and the deviation of the surrogate distribution. After the above normalization, we compared MIs between conditions with the same method as the PPC.

#### Within-Frequency Phase Synchronization Analysis for EEG Data

Oscillations at low frequency may mediate functional connectivity between distant regions, while high-frequency oscillations are considered to reflect oscillatory patterns of local activity (Buzsaki, 2009). If the γ-ERS shows the cross-frequency coupling with alpha rhythm, the local activity at the gamma rhythm is considered to be transferred to another region via alpha-rhythm oscillation. We analyzed phase synchronization in the alpha band between channels. We calculated the debiased weighted phase lag index (dwPLI) to reduce the effect of spurious synchrony caused by volume conduction from a common source (Vinck et al., 2011). The dwPLI is computed with following formulas:

$$d\,w\,P\,L\,I=\frac{\sum_{j=1}^{N-1}\sum_{k=j+1}W_{j,k}\,d\,\left(X_{j},X_{k}\right)}{\sum_{j=1}^{N-1}\sum_{k=j+1}W_{j,k}}$$

$$W_{j,k}=\left|I\,m\,\left\{X_{j}\,I\,m\,\left\{X_{k}\right\}\right\}\right|$$

$$d\,\left(X_{j},X_{k}\right)=s\,g\,n\,\left(I\,m\,\left\{X_{j}\right\}\right)\,s\,g\,n\,\left(I\,m\,\left\{X_{k}\right\}\right)$$

where *X*_*k*_ is the wavelet cross-spectrum of two different electrodes at time point k, and *Im*{*X*_*k*_} is the imaginary part of the *X*_*k*_. *N* is the number of data points within the calculation window. To evaluate the dwPLI change, we compared the dwPLIs between the target time-bin and the baseline (Kawasaki et al., 2010). The evaluation was conducted with the permutation test alike the cross-frequency coupling (see section “Cross-Frequency Coupling Analysis for EEG Data”). The statistical comparison was done with *z*-value from the right-sided Wilcoxon signed rank test.

#### ERS/D-Based PPI Analysis for NIRS Data

**FIGURE 3:**
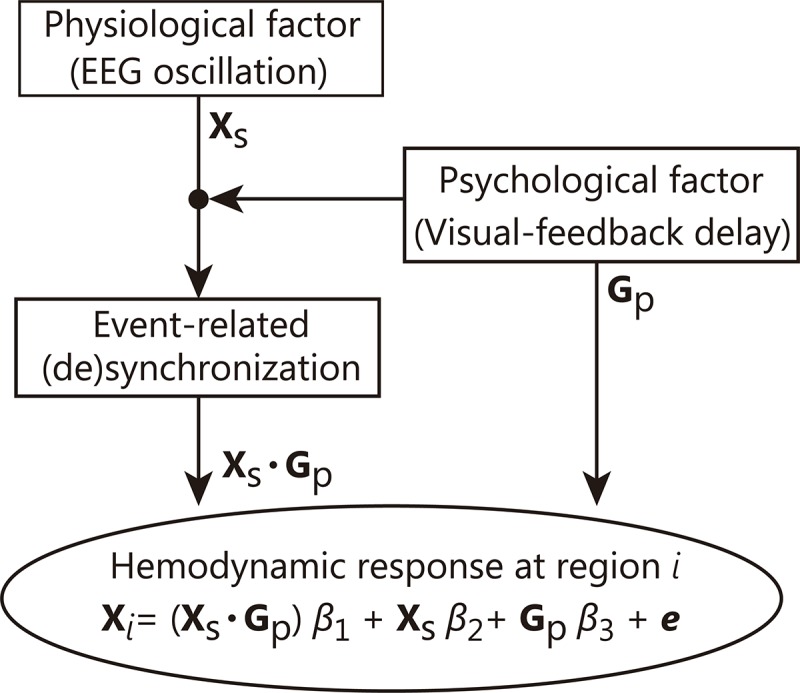
Schematic chart of our ERS/D-based PPI analysis. In this technique, the hemodynamic response (explained variable X*_*i*_*) in a region *i* is estimated as a superposition of impulse responses. The regression coefficient is calculated so that the error (*e*) between the estimated hemodynamic response (X*_*i*_*) and the observed hemodynamic response is minimized. This analysis can be interpreted as estimating the area where the contribution from a certain ERS/D is increased by an event (psychological or experimental factor).

We primarily analyzed oxyHb because it is considered to be the most sensitive parameter of change in regional cerebral blood flow and is most strongly correlated with the blood oxygen level dependent (BOLD) signal among the three NIRS parameters (oxyHb, deoxyHb, and totalHb) (Hoshi et al., 2001; Strangman et al., 2002). The oxyHb data were preprocessed using a zero-phase filter that ranged from 0.02 to 2 Hz. To investigate brain areas in which activity was related to EEG frequency power during the visual feedback delay, we developed a new ERS/D-based PPI analysis that extends the original psychophysiological interaction (PPI) analysis (Friston et al., 1997) used to analyze hemodynamic responses. The original PPI analysis is based on following general linear model:

$$X=\left(X_{s}\times G_{p}\right)\,\beta_{1}+X_{s}\,\beta_{2}+G_{p}\,\beta_{3}+e$$

where **X** is the explained variable (i.e., hemodynamic response at a target region; in our performed analysis, the changes in oxyHb concentration at each NIRS channel registered on the cortical surface), β_*X*_ are regression coefficients, the **X*_*s*_*** regressor is the hemodynamic response at a seed region, the **G*_*p*_*** regressor is a psychological variable (in our analysis, whether or not the visual feedback was delayed), and ***e*** is the error term. The PPI regressor (**X*_*s*_*** × **G*_*p*_***) reflects the interaction between the neural activity and the psychological factor. In the ERS/D-based PPI analysis, we replaced the hemodynamic response at seed region (**X*_*s*_***) with a hemodynamic response model correlated with the ERS or ERD (Figure 3). This data-driven hemodynamic response model was constructed by convolving the hemodynamic response function [a Gaussian kernel of dispersion of 4-s full-width half-maximum, as in Shimada et al. (2005) and Shimada and Hiraki (2006)] with the ERS/D. In this study, since we found γ-ERS that was specific to visual feedback delay at the C1 electrode, we used that γ-ERS to generate a data-driven hemodynamic response model (see section “Time-Frequency Analysis for EEG Signals”). Based on a report that hemodynamic responses start within 500 ms of neural activity (Masamoto and Kanno, 2012), we inserted an additional 500-ms delay to the rise time of the hemodynamic response during model building. The **G*_*p*_*** regressor was a box-car function indicating the Delayed condition (+ 1) or Non-delayed condition (−1) convolved with the Gaussian kernel. Hence, in this study, the region where the ERS/D-based PPI regressor (**X*_*s*_*** × **G*_*p*_***; psycho-physiological interaction) is relevant is the region where a specific fluctuation of ERS/D influenced its activity only under the Delay condition.

The psychophysiological interaction in the original PPI analysis can be interpreted to mean that (1) the contribution of one area (seed region) to another area (target region) has changed with the psychological context or (2) the seed region modulates the responsiveness of the target region to the psychological factor (Friston et al., 1997). In our method, we replaced the physiological factor from an activation of a seed region to the specific information processing represented by ERS/D rich in time information. Thus, our technique focuses on “how” the target region is affected rather than “from where.”

#### GLM Analysis for NIRS Data

To confirm that the activation areas were related to the reaching movements, we conducted a GLM analysis for the NIRS signal alone based on the following model:

$$X=X_{\mathrm{𝐷𝑒𝑙𝑎𝑦}}\,\beta_{1}+X_{\mathrm{𝑁𝑜𝑛}-\mathrm{𝑑𝑒𝑙𝑎𝑦}}\,\beta_{2}+e$$

**X*_*Delay*_*** and **X*_*Non–delay*_*** regressors are hemodynamic response models for each condition represented by a box car function convolved with the Gaussian kernel. We also conducted a standard PPI analysis to determine whether any regions exhibited enhanced functional connectivity with the IPL during visual feedback delay.

## Results

### Behavior

**FIGURE 4:**
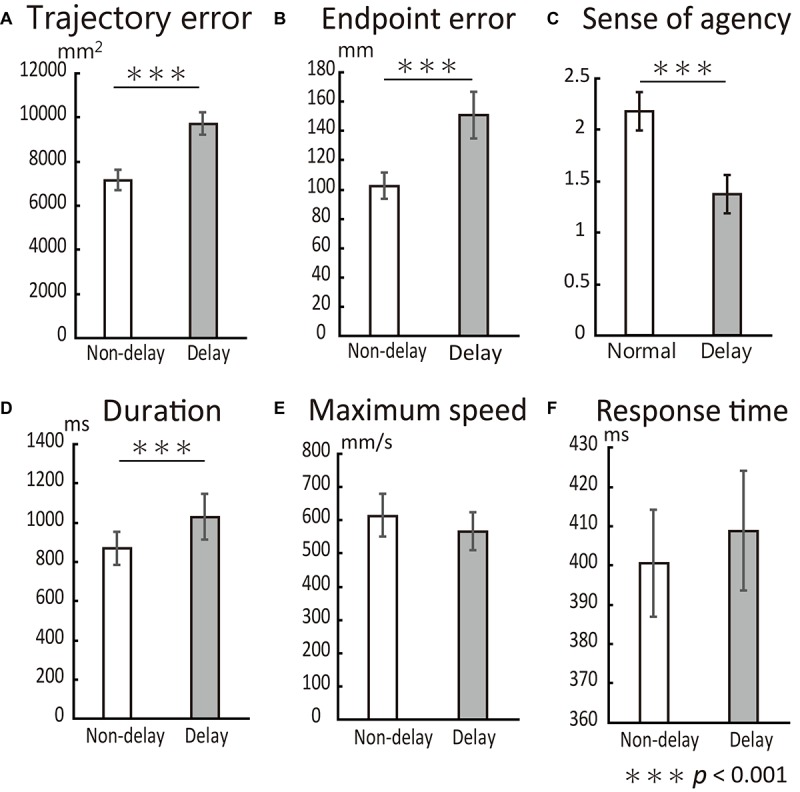
Behavioral results. All error bars represent the mean *SE.* **(A)** Trajectory error was significantly increased by the delay in visual feedback. **(B)** Endpoint error was also significantly increased by the delay. **(C)** SoA score dropped with the delay. **(D)** The duration of reaching movements was significantly larger in the Delay condition than in the Non-delay condition but did not affect task performance. Neither maximum reaching speed **(E)** nor response time **(F)** differed between conditions.

Figure 4 depicts the indices of reaching performance and the SoA scores. The trajectory error for the Delay condition was significantly larger than that for the Non-delay condition (*t*_(__13__)_ = 4.69, *p* < 0.001), as was the endpoint error (*t*_(__13__)_ = 4.45, *p* < 0.001). SoA scores were significantly smaller in the Delay condition than in the Non-delay condition (*t*_(__13__)_ = −4.46, *p* < 0.001). Hence, we confirmed that performance errors increased, and the sense of agency decreased as a result of the delay in visual feedback. Furthermore, reaching movements in the Delay condition took more time to perform (Delay: 1028.5 ± 115.0 ms (mean ± *SE*); Non-Delay: 867.4 ± 84.6 ms; *t*_(__13__)_ = 4.48, *p* < 0.001), while maximum velocity (Delay: 566.1 ± 57.4 mm/s; Non-Delay: 613.1 ± 65.2 mm/s; *t*_(__13__)_ = −1.60, *p* = 0.13) and response time (Delay: 408.8 ± 1.5 ms; Non-Delay: 400.5 ± 1.4 ms; *t*_(__13__)_ = 2.03, *p* = 0.063) did not differ between conditions. These results indicate that the change in performance was caused by the delay in visual feedback and that it was not a consequence of a speed-accuracy tradeoff.

### Time-Frequency Analysis of EEG Signals

**FIGURE 5:**
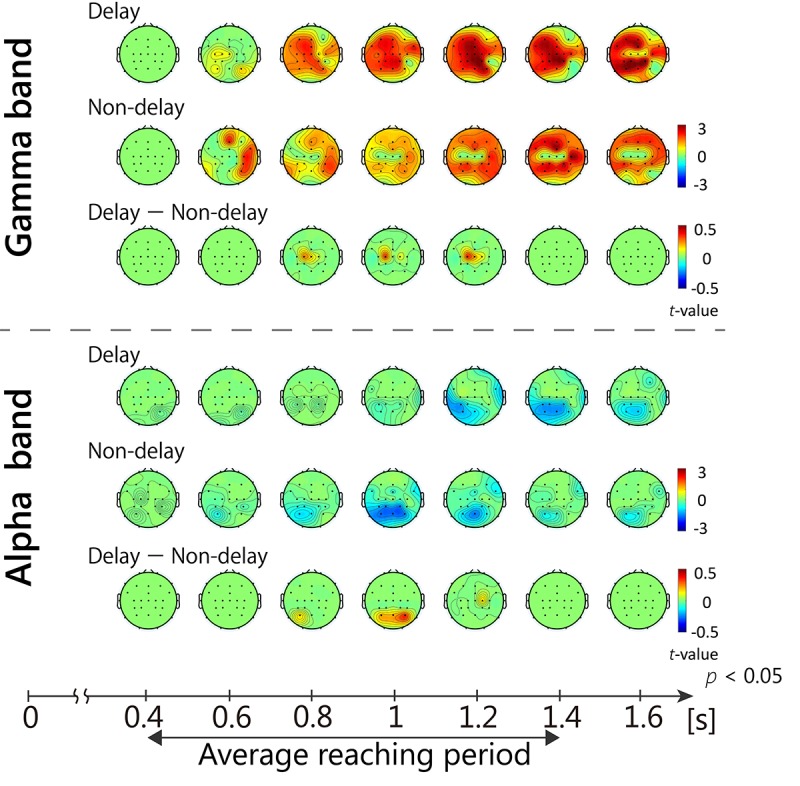
Sequential topological maps of EEG power. γ-ERS and α-ERD during movement differed significantly between conditions (*p* < 0.05, FDR corrected). The γ-ERS specific to the Delay condition occurred at the central area, while α-ERD occurred around the parietal area.

Time-frequency analysis revealed a characteristic change in the gamma-band power range (31–47 Hz). Figure 5 shows sequential topological activation maps of the increase in EEG power. The ERS in the gamma band oscillation (γ-ERS) occurred during reaching movements and was greater in the Delay condition at the central area (top 3 rows of Figure 5, *p* < 0.05). At the C1 electrode, significant differences between conditions were confirmed during the 0.8 to 1.2 s time bin (*p* < 0.05, FDR corrected). Additional investigation for other frequency bands showed significant alpha (8–13 Hz) power attenuation at the parietal region (α-ERD; bottom three rows of Figure 5). During movement (0.8 to 1.0 s), the α-ERD at the P3 electrode in the Non-Delay condition was more negative than that in the Delay condition (*p* < 0.05, FDR corrected). We found no correlation between EEG fluctuations (γ-ERS and α-ERD) and behavioral performance (*p* > 0.05). Thus, specific fluctuations in γ and α power occurred during movement when visual feedback was delayed, although they were not related to performance or the sense of agency in controlling the movement.

We also conducted current-source density estimation using standardized low-resolution brain electromagnetic tomography (sLORETA) (Pascual-Marqui, 2002, 2007). However, the analysis did not reveal any statistically significant origin of the EEG at the time-bin when the γ-ERS/α-ERD indicated a significant difference between conditions.

### Cross-Frequency Coupling of EEG

**FIGURE 6:**
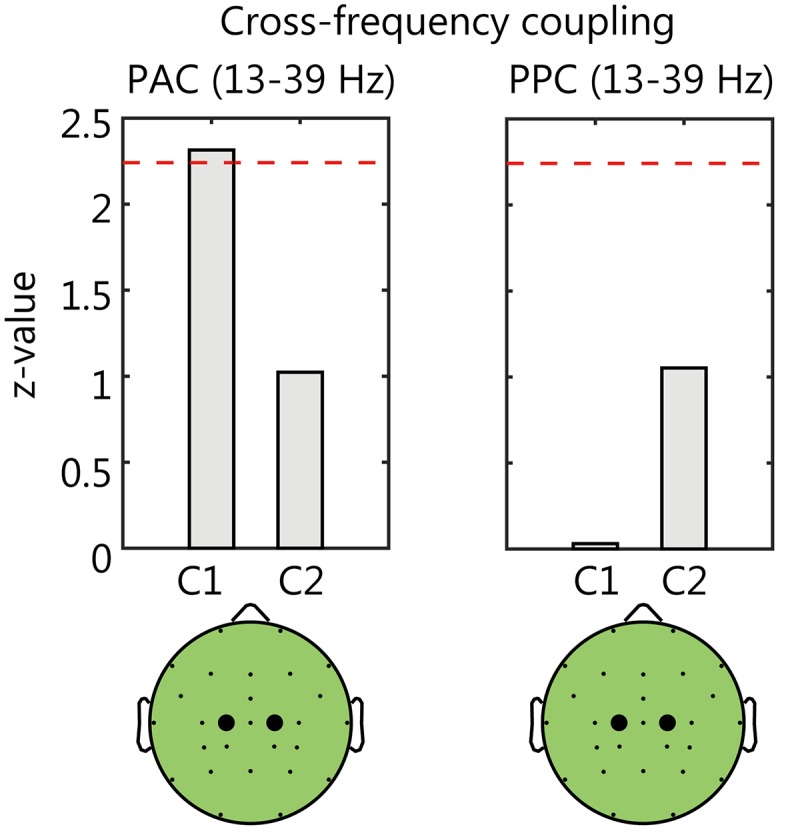
The results of cross-frequency coupling analysis between the alpha- and gamma-rhythms. The comparison between Delay and Non-delay conditions of phase-amplitude coupling (PAC, **left**) and phase-phase coupling (PPC, **right**). The dashed lines in each panel denote the threshold value (*p* < 0.05, Bonferroni corrected). At the C1 electrode, the PAC in the Delay condition was significantly larger than that in the Non-Delay condition.

We analyzed the cross-frequency coupling at the C1 electrodes where the Delay-condition specific γ-ERS was most remarkable. We also analyzed the coupling at the C2 electrode for comparison. The results showed that the relationship between the alpha- and gamma-rhythms at C1 electrode (Figure 6). The phase of alpha-rhythm (13 Hz) synchronized with the amplitude envelope of gamma rhythm (39 Hz) (*z* = 2.31, *p* < 0.05, Bonferroni corrected). This coupling was not found at the C2 electrode (*p* > 0.05). On the other hand, both C1 and C2 electrodes showed no phase-phase coupling between the alpha- and gamma-rhythms (*p* > 0.05). From the above, the γ-ERS at C1 under the visual feedback delay was associated with the phase of alpha rhythm.

### Within-Frequency Phase Synchronization

**FIGURE 7:**
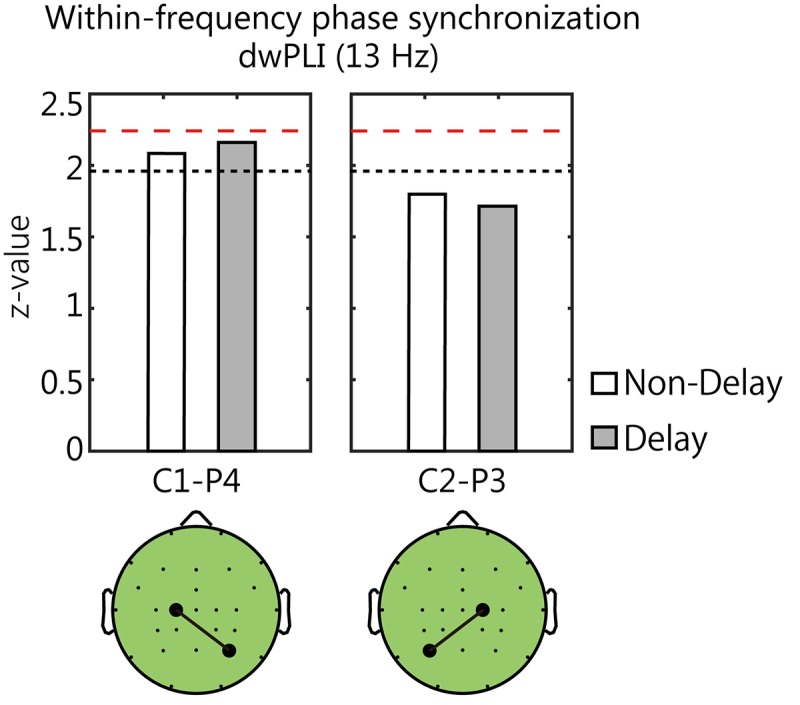
The phase synchronization of alpha-rhythm between the central and the parietal area. The black dotted lines and red dashed lines in each panel denote the threshold values (*p* < 0.1 and *p* < 0.05, respectively). The increase in synchrony was marginally significant between the **left** central electrode (C1) and the **right** parietal electrode (P4) in either condition (*p* < 0.1, Bonferroni corrected).

To investigate whether the γ-ERS at C1 electrode was integrated into the right parietal area via alpha-rhythm, we analyzed phase synchronization between C1 and P4 electrodes (Figure 7). We also investigated the phase synchronization between C2 and P3 electrodes for the comparison. As for the C1-P4 pair, the increases in synchrony from the baselines were marginally significant in both conditions (*z* = 2.16 and *p* = 0.061, *z* = 2.08 and *p* = 0.074, respectively; Bonferroni corrected). On the other hand, opposite C2-P3 pair showed no significant increase in synchrony in either of condition (*p* > 0.05). So, it was shown that the alpha-rhythm oscillations at C1 and P4 electrodes tend to be synchronized during movement.

### ERS/D-Based PPI Analysis

**FIGURE 8:**
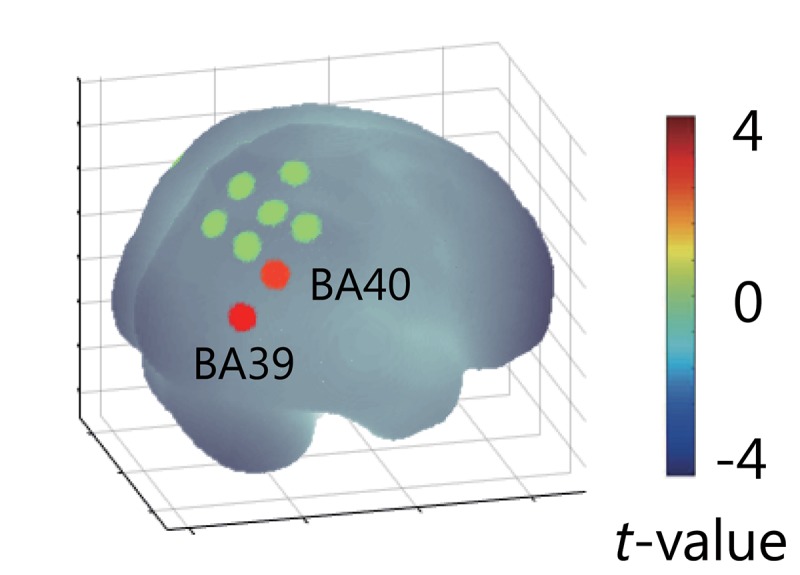
Results of ERS/D-based PPI analysis. The result showed activation of right angular and supramarginal gyri, which was explained by the γ-ERS that occurred when visual feedback was delayed (*p* < 0.05). This delay-specific activity in the right IPL could not explained by α-ERD.

The time-frequency analysis for EEG signals showed that the largest and most remarkable γ-ERS occurred at the C1 electrode when the visual feedback was delayed. To investigate whether the parietal regions would be affected by the γ-ERS in the Delay condition, we integrated the γ-ERS (C1 electrode, 39 Hz as the most prominent frequency, 0.8 to 1.2 s time-bin) into the ERS/D-based PPI analysis. The target region (region of interest, i.e., NIRS channels for which changes in oxyHb were hypothesized to be explained by delay-specific γ-ERS and experimental factors) was set to the bilateral parietal lobe. The ERS/D-based regressor (**X*_*s*_*** × **G*_*p*_***) showed activation of the right angular gyrus [Montreal Neurological Institute (MNI) coordinates: (57, −72, 16), *t*_(__13__)_ = 3.17, *p* < 0.05, FDR corrected] and supramarginal gyrus [MNI coord.: (61, −59, 16), *t*_(__13__)_ = 2.61, *p* < 0.05, FDR corrected] (Figure 8). When we analyzed the ERS/D-based regressor using α-ERD, the results did not explain the activity in the parietal region, although the timing of the fluctuations in α-ERD that were specific to the delay were mostly the same as those obtained when we used γ-ERS. Furthermore, no hemodynamic response could be explained by the γ-ERS-based regressor (**X*_*s*_***) in the NIRS measurement range (*p* < 0.05, FDR corrected). Thus, although no activated areas correlated with the γ-ERS in any condition, the γ-ERS during the Delay condition explained the right-IPL responses to the delayed visual feedback. As with the ERS/D analysis, IPL activity did not correlate with the behavioral data (*p* > 0.05). These results indicate that the right IPL responded in real-time to multisensory inconsistency during reaching movements, and that, despite the absence of a correlation with performance or the judgment of agency of the movements, gamma rhythms contributed to this process.

### NIRS Signal Analysis

**FIGURE 9:**
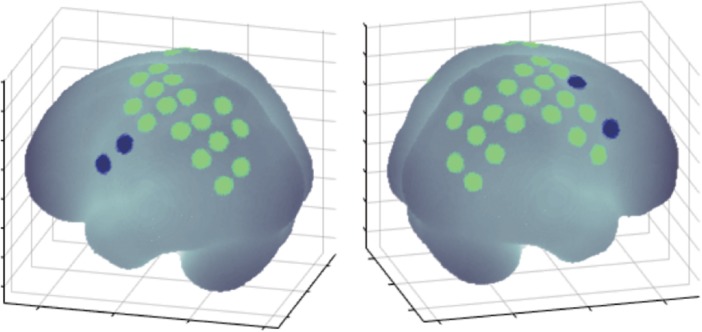
Results of the LM analysis. The areas identified by green dots represent activation areas common to both the Delay and Non-delay conditions. Several regions (blue dots) were activated only during the Delay condition.

The GLM analysis of the NIRS signals showed significant wide-spread activation at the sensorimotor area and the parietal lobe for each condition (*p* < 0.05, FDR corrected). Activation areas that were shared between the conditions were most frequently observed during single trials with short movements, and in some trials, premotor area activation was observed only during the Delay condition (Figure 9). Furthermore, the PPI analysis showed no functional connectivity with the IPL (*p* > 0.05). These results indicate that regardless of whether the delay was present, the IPL was activated equally in relation to the reaching movement. Further, they show that the regions associated with responses specific to delays in visual feedback (i.e., the IPL) could not be confirmed from hemodynamic responses alone.

## Discussion

We investigated whether the IPL responds to multisensory inconsistencies online during reaching movements by simultaneously recording EEG-NIRS signals during a visual feedback-delay task. Time-frequency analysis of EEG signals revealed power fluctuations specific to delayed visual feedback during movement. Among these fluctuations, γ-ERS explained the hemodynamic responses in the right IPL only when visual feedback was delayed. This real-time response in the IPL was absent in isolated analysis of NIRS signal. Although the single modality analysis with EEG signals showed that the gamma-rhythm activity under Delay condition could be associated with the parietal region, EEG’s spatial information was too rough to mention the IPL. The real-time respond in the IPL could only be detected using the ERS/D-based PPI analysis that integrated the two types of information. However, while behavioral performance was affected by the delayed feedback, it was not correlated with any physiological indices.

The main feature of our ERS/D-based PPI analysis is that it is driven only by event-related EEG information, rather than by the entire EEG time series. The hemodynamic response associated with neural activity has been reported by neurophysiological studies in animals (Roy and Sherrington, 1890; Hoshi et al., 2001; Harrison et al., 2002; Kuramoto and Kanno, 2012; Nemoto et al., 2012). In the human studies, the relationship between electrical activity of brain and its hemodynamic response has been reported by the EEG and fMRI/NIRS signals (Horovitz and Gore, 2004; Takeuchi et al., 2009; Näsi et al., 2010; Engell et al., 2012; Fuglø et al., 2012; Zama and Shimada, 2015). In previous human studies, researchers have conducted GLM analyses with hemodynamic responses modeled by convoluting the hemodynamic response function and the oscillation of EEG power (Rosa et al., 2010; Sato et al., 2010; Engell et al., 2012; Fuglø et al., 2012; Onojima et al., 2017). Fuglø et al. (2012) examined the ability of various EEG-based regressors to estimate hemodynamic responses to visual stimuli. They reported that including regressors based on EEG frequency power was effective when investigating brain activity that fluctuates in ways that cannot be modeled by boxcar regressors that are based on psychological factors. In particular, some studies have demonstrated that changes in EEG gamma-band power can explain hemodynamic responses observed in fMRI (Engell et al., 2012; Magri et al., 2012; Mizuhara, 2012). Although these previous studies suggested that data-driven regressors are useful in investigating brain activity, they generally assumed that information included in the time series of frequency power is uniform. This is the assumption that a particular frequency reflects a particular (unitary) function. However, EEG rhythms are known to be involved in numerous functions. For example, the gamma rhythm has been implicated in movement (Crone et al., 1998; Pfurtscheller et al., 2003), attention (Womelsdorf and Fries, 2007), multisensory integration (Kaiser et al., 2005; Sakowitz et al., 2001, 2005; Senkowski et al., 2005; Kanayama et al., 2007, 2009), and conscious perception (Melloni et al., 2007). This also applies to the relationships between brain regions and cognitive function. With respect to the IPL, reports indicate that it is involved not only in multisensory inconsistency (Farrer et al., 2003, 2008; Shimada et al., 2005; Balslev et al., 2006), but also in the sensorimotor processing (Mattingley et al., 1998), motor organization based on a specific action goal (Bonini et al., 2010), motor perception (Naito and Ehrsson, 2006), formation of body image based on multisensory integration between visual and somatosensory information (Leinonen and Nyman, 1979), and action intention (Fogassi et al., 2005; Fogassi and Luppino, 2005; Desmurget and Sirigu, 2009). Thus, linearly regressing observed hemodynamic responses during movement with time-series EEG power data is unreasonable because the correspondence between an EEG rhythm and the source changes in a fluid way. Therefore, we focused on event-related (de)synchronization such as γ-ERS to limit interpretation of the EEG rhythm. Simply put, ERS can be regarded as an increase in the population of neurons that synchronously fire at a specific rhythm. Hence, based on a report that hemodynamic responses follow neural activity (Masamoto and Kanno, 2012), modeling hemodynamic responses using event-related EEG-power fluctuations appears to be reasonable. By this modeling, we could detect the IPL response correlated with γ-ERS under the Delay condition. The GLM analysis for NRIS signals showed the IPL activation, but the difference between conditions could not detected with the temporal resolution of NIRS in our experimental task. On the other hand, synchronization analysis of EEG signals showed the marginally phase synchronization of alpha-rhythm oscillations between C1-P4 electrodes during movement, and the coupling between the phase of alpha-rhythm oscillation and the amplitude of gamma-rhythm oscillation at C1 electrode under the Delay condition. These results of EEG data suggest that the gamma-rhythm activity at C1 electrode is involved in the activity at C4 electrode via alpha rhythm. The ERS/D-based PPI analysis was superior in that it was able to integrate these results of each modality.

In this study, we conducted ERS/D-based PPI analysis using the γ-ERS with a particular focus on the size of the time bin and the degree to which the power was significantly enhanced under the Delay condition. That is, in terms of the physiological factor in PPI analysis, we focused on “information processing accompanied by γ-ERS specific to visual feedback delay” instead of “hemodynamic response in the seed region.” Accordingly, our results by the ERS/D-based PPI analysis can be interpreted to mean that (1) the contribution of gamma rhythm activity from a certain region to the IPL increases in the presence of a visual feedback delay, or (2) the response of the IPL during reaching movement in the presence of a visual feedback delay is modulated by information processing accompanied with γ-ERS that is specific to the delay. However, we found no region in which hemodynamic responses were correlated with gamma rhythm activity, regardless of the task condition. Furthermore, although there were regions in which significant hemodynamic responses were observed in the Delay condition only, the PPI analysis of NIRS signals revealed no regions that showed connectivity with the IPL under the Delay condition. From these results, it appears that there was no source of γ-ERS, at least within the range of NIRS measurement. In addition, since no significant results were obtained from EEG source estimation, it is not possible to determine which of the two above interpretations is more likely. This may be a limitation of ERS/D-based PPI analysis using EEG-NIRS.

The EEG gamma rhythm is thought to be involved in information integration. γ-ERS was reported to occur when participants perceived human faces from black-and-white images by integrating information such as edges (Rodriguez et al., 1999). Other studies have reported that γ-ERS relates to multisensory integration (Sakowitz et al., 2001, 2005; Kaiser et al., 2005; Senkowski et al., 2005; Kanayama et al., 2007, 2009). Kanayama et al. (2007) reported that γ-ERS was correlated with multisensory integration processing, becoming larger when multimodal information was consistent compared with when it was inconsistent. However, the role of gamma rhythms in these reports is not consistent with our results. We found that the larger γ-ERS only occurred when visual feedback was delayed. Thus, the γ-ERS in this study may reflect another function that is unrelated to multisensory integration because the PPI analysis of the NIRS signals revealed no connectivity between the IPL and other sensory association areas that are thought to be implicated in multisensory integration (Lewis and Van Essen, 2000). The γ-ERS in the present study occurred about 400 ms after movement onset (i.e., when the body image belatedly started to move), and not immediately after. Therefore, we suggest that the γ-ERS reflects a process relating to “prediction errors” rather than “multisensory integration.” From the viewpoint of predictive coding (Friston, 2010), gamma band activity is considered to reflect prediction errors (von Stein et al., 2000; Arnal et al., 2011; Arnal and Giraud, 2012; Bastos et al., 2012; Bauer et al., 2014; van Pelt et al., 2016; Gillies et al., 2019). Arnal et al. (2011) reported that gamma activity in the STS is correlated with cross-modal inconsistency. Another study reported that bottom-up signals appear to be propagated from the STS to the TPJ via gamma rhythms and that gamma power increases during sensory prediction errors (van Pelt et al., 2016). Consistent with our results, Völker et al. (2018) showed that the error signal was related to γ-ERS at centrally located electrodes. These previous reports that gamma band activity reflects bottom-up propagated prediction error are consistent with the results of our γ-ERS and ERS/D-based PPI analysis. Based on the above, our results suggest that the sensory prediction error, that occurred during the reaching movement under the visual feedback delay condition, is involved in the activity of the IPL in real-time.

Previous studies have reported activation of the IPL, especially the right angular gyrus, when participants attribute the agency of actions to others even though they themselves commit the movement (Spence et al., 1997; Farrer and Frith, 2002; Farrer et al., 2003, 2008). This pattern of activation has been observed for both voluntary and involuntary movement (Shimada et al., 2005; Balslev et al., 2006). Hence, the angular gyrus is considered to play a role in detecting the movement of others. In these studies, however, participants observed continuous movements while being exposed to multisensory inconsistency, and brain activity was measured over a long period from movements to judgment of agency. The temporal resolution of the recording methods used in these studies was insufficient to separate brain activity related to multisensory inconsistency from that related to judgments of agency. Here, EEG-NIRS data showed that the timing of activity in the right IPL mirrored the input of prediction error during individual reaching movements. Considering that IPL activity was not correlated with movement performance or the SoA score, the real-time responses could reflect bottom-up processing of the “feeling of agency” at the sensorimotor level rather than a direct representation of the retrospective “judgment of agency” (Synofzik et al., 2008). Indeed, IPL activities are not likely to be irrelevant to judgments of agency. Since judgment of agency, as a higher-level cognitive action compared with feelings of agency, is affected not only by sensory prediction errors but also by various factors such as intention, thought, and contextual cues, it may not be possible to express judgment of agency as a simple regression of IPL activity. However, it would be very difficult to effectively consider all of the factors that affect judgment of agency, and this problem hinders elucidation of the role of the SoA in subjective consciousness. Interpreted cautiously, the results of this study suggest that the IPL is affected in real-time by multisensory inconsistency processing. Although further investigation is necessary, IPL activity that responds to prediction errors may reflect processing related to understanding motor intention. When Desmurget and Sirigu (2009) directly stimulated the right angular gyrus, patients reported motor intention. Furthermore, increasing the stimulation intensity caused motor illusion. The stimulated area has also been reported to work as a mirror neuron system (MNS) for understanding the movement intention of others (Nishitani and Hari, 2002; Fogassi et al., 2005; Rizzolatti et al., 2006). The integration and comparison of estimated intention from images of one’s own body and images of the bodies of others may be helpful in distinguishing oneself from others.

In this experiment, in addition to the γ-ERS, we found significant α-ERD around parietal region. The α-ERD relating to the reaching movement has been reported to occur around the parietal region (Fumuro et al., 2015). The ERD at alpha-band occurs in relation to various movements and the α-ERD in the central region is called mu-suppression (Pfurtscheller and Neuper, 1994; Leocani et al., 1997; Cochin et al., 1999; Ramoser et al., 2000; Muthukumaraswamy et al., 2004; Pineda, 2005). Previous studies have reported that the magnitude of mu-suppression is modulated by various factors such as task complexity (Boiten et al., 1992; Dujardin et al., 1995) and feelings of self-control (Wen et al., 2017). Wen et al. (2017) investigated mu-suppression under conditions in which subjects were in control of a cursor on a screen or were not in control. The researchers found that the magnitude of mu-suppression became significantly larger during the experience of control, regardless of whether the participants actually had control of cursor movement. In our experiment, we found stronger α-ERD in the Non-delay condition than in the Delay condition, suggesting that participants more clearly felt a sense of control when there was no delay in visual feedback. However, the ERS/D-based PPI analysis indicated that the α-ERD had no influence on NIRS signals. While ERD is considered to reflect a decrease in neural activity, enhanced alpha power often signifies an idling state (Pfurtscheller et al., 1996). Thus, the conditions that would lead to a significant difference in α-ERD between conditions are complex, making it difficult to interpret the functional meaning. As a result, modeling hemodynamic response by ERS appears to be preferable to modeling by ERD. Finally, it is also possible that no brain regions were affected by α-ERD in the NIRS measurement range. Further verification is needed regarding ERS/D-based PPI analysis using ERD.

One of concerns of this study is the contamination of EEG signals by electromyography signals, and NIRS signals by scalp blood flow and head motion. Although we made efforts to reduce the contamination by the instruction to participants and using chin rest, the contamination of those signals is considered to be occur more or less, so it should be noted that there is a possibility that our results were provided by the effect of contamination. However, if the results by the ERS/D-based PPI analysis were produced by the contamination of NIRS signals due to head-motion derived changes in signals and scalp blood flow, similar results should be obtained in other areas. Hence, we considered that the effects of scalp blood flow and head motion on NIRS signals were sufficiently small.

In conclusion, we proposed an ERS/D-based PPI analysis that estimates fluctuations in NIRS signals associated with the ERS/D of EEG signals. This method is beneficial to investigate whether the IPL responds to the multisensory inconsistency caused by visual feedback delay during controlling a reaching movement. Our results suggest that, although the right IPL receives online prediction error signals, the IPL online response to the prediction error does not directly represent the conscious experience of movement agency.

## Ethics Statement

This study was carried out in accordance with the recommendations of the principles and guidelines of the Declaration of Helsinki with written informed consent from all subjects. All subjects gave written informed consent in accordance with the Declaration of Helsinki. The protocol was approved by the ethics committee of the School of Science and Technology, Meiji University.

## Author Contributions

TZ, YT, and SS designed the experiment, and reviewed and approved the final manuscript. TZ and YT setup and conducted the experiment, and analyzed the data. TZ and SS wrote the manuscript.

## Conflict of Interest Statement

The authors declare that the research was conducted in the absence of any commercial or financial relationships that could be construed as a potential conflict of interest. The reviewer TH declared a shared affiliation, though no other collaboration, with one of the authors TZ to the handling Editor.
